# To live free or being a parasite: The optimal foraging behavior may favor the evolution of entomopathogenic nematodes

**DOI:** 10.1371/journal.pone.0298400

**Published:** 2024-03-13

**Authors:** Víctor Trejo-Meléndez, Jorge Contreras-Garduño

**Affiliations:** 1 Posgrado en Ciencias Biológicas, Universidad Nacional Autónoma de México, Coyoacán, Mexico City, CDMX, Mexico; 2 Escuela Nacional de Estudios Superiores, Unidad Morelia, UNAM., Morelia, Michoacán, Mexico; 3 Instituto for Evolution and Biodiversity, University of Münster, Münster, Germany; Chiang Mai University Faculty of Agriculture, THAILAND

## Abstract

Facultative parasites can alternate between a free-living and a parasitic existence to complete their life cycle. Yet, it remains uncertain which lifestyle they prefer. The optimal foraging theory suggests that food preferences align with fitness benefits. To test this hypothesis, we investigated the facultative parasite nematode *Rhabditis regina*, assessing its host preference and the associated benefits. Two experiments were conducted using wild nematode populations collected from *Phyllophaga polyphylla*, their natural host. In the first experiment, we used a behavioral arena to assess host preference between the natural host and two experimental hosts: *Spodoptera frugiperda* which is an alternative host and dead *Tenebrio molitor*, which simulates a saprophytic environment. In the second experiment, we subjected wild nematodes to "experimental evolution" lasting 50 generations in *S*. *frugiperda* and 53 generations in *T*. *molitor* carcass. We then compared life history traits (the size, survival, number of larvae, and glycogen and triglycerides as energy reserves) of dauer larvae with those nematodes from *P*. *polyphylla* (control group). We found a significant preference for *P*. *polyphylla*, which correlated with higher values in the nematode’s life history traits. In contrast, the preference for *S*. *frugiperda* and the saprophytic environment was lower, resulting in less efficient life history traits. These findings align with the optimal foraging theory, as the nematode’s parasitic preferences are in line with maximizing fitness. This also indicates that *R*. *regina* exhibits specificity to *P*. *polyphylla* and is better adapted to a parasitic lifestyle than a free-living one, suggesting an evolutionary pathway towards parasitism.

## Introduction

The decision to embrace an independent lifestyle or adopt a parasitic existence constitutes a fundamental aspect of an organism’s life strategy. This choice is intricately shaped by a combination of ecological factors and evolutionary pressures. Facultative parasites exhibit a unique ability to alternate between resources, thereby completing their life cycles [1]. This adaptability categorizes them as nascent stages within purely parasitic lineages. Nevertheless, the existence of a distinct preference for either a parasitic or free-living lifestyle, and the factors influencing this dietary inclination, remain uncertain. Within this context, the theory of optimal foraging postulates that behaviors associated with food acquisition should yield more benefits than costs in terms of fitness [2–4]. This implies that if a facultative parasite’s life history traits, encompassing growth, survival, and reproduction patterns, are enhanced by exploiting a host, a preference for parasitism over resource acquisition akin to free-living organisms should prevail. While food preferences have been explored in parasitoids, vectors, and selected examples of trematodes [5–10], one of the most diverse groups of parasites, nematodes, remains largely unexplored. Existing studies have predominantly concentrated on host-related factors influencing preferences, such as host size, susceptibility (resistance to infection), density, or diversity. However, the examination of this food preference within the optimal foraging framework is yet to be thoroughly investigated. In this regard, natural selection favors the recognition and exploitation of hosts that optimize parasite fitness [4, 12], thereby facilitating the evolution of optimal host preference in response to these evolutionary imperatives. Despite numerous studies demonstrating the influence of host-related factors, such as host species, on a parasite’s life history traits [13–16], this area of research remains relatively uncharted concerning facultative parasites. Consequently, an exploration of the evolution of parasitism within the framework of optimal foraging would contribute valuable insights into the evolutionary and ecological factors steering this transition in facultative parasites.

Nematodes, owing to the abundance of species exhibiting facultative parasitic behavior, serve as ideal subjects for testing optimal foraging theory [17]. An intriguing subset within the phylum Nematoda is the entomopathogenic nematodes (EPN), renowned for their parasitic relationships with insects facilitated by mutualistic bacteria [18–20]. While these nematodes have demonstrated the capacity to exploit saprophytic environments, those from the families Steinernematidae and Heterorhabditidae are generally considered obligate parasites [11–13]. Numerous studies have supported this classification by unveiling specific adaptations associated with a parasitic lifestyle [11, 14, 15]. For instance, EPN larvae exhibit chemotaxis behavior toward CO_2_, pheromones, or host-specific odors, promoting effective foraging behavior [16–18]. Despite reports of entomopathogenicity in nematodes from other families [19–22], a lack of comprehensive studies has relegated them to the status of non-authentic entomopathogens or facultative entomopathogenic organisms [13]. *Rhabditis regina* is a noteworthy example, acting as an entomopathogen of *Phyllophaga polyphylla* larvae while also demonstrating saprophytic tendencies under laboratory conditions [19, 23]. Its consistent prevalence on an annual host like *P*. *polyphylla* supports a parasitic inclination, yet its sustained survival in the field suggests the utilization of alternate hosts or resources to contend with host seasonality [24].

Recognizing that exploiting diverse hosts or environments can be advantageous against host seasonality, we designed experiments to evaluate *R*. *regina* host preference and the evolution of its life history traits across three distinct hosts: *P*. *polyphylla*, *S*. *frugiperda*, and *T*. *molitor* (in the presence of deceased insects). Our hypothesis rested on the assumption that *R*. *regina* employs an optimal foraging strategy, one that yields more benefits than costs in terms of fitness. Our prediction was that *R*. *regina* would exhibit a preference for *P*. *polyphylla* due to its positive impact on adult size, longevity, larvae production, and dauer larvae energy reserves compared to alternative hosts and environments. The results substantiated our hypothesis and predictions. *R*. *regina* unmistakably displayed a preference for live hosts over deceased ones, with *P*. *polyphylla* emerging as the most favored. This preference correlated with significantly enhanced life history traits observed in the natural host. The skew towards a parasitic existence and the associated benefits strongly suggests that *R*. *regina* is more adept at an entomopathogenic lifestyle than a free one. This study furnishes compelling evidence that the concept of optimal foraging is pertinent to understanding the evolution of parasitism, providing valuable insights into the evolutionary and ecological trajectory of facultative parasites.

## Materials and methods

### Nematodes and hosts

#### *Rhabditis regina* and *Phyllophaga polyphylla*

The natural host, *P*. *polyphylla*, was collected from corn fields in Jerecuaro, Guanajuato, Mexico (20° 08′ 58″ N, 100° 30′ 34″ W). The process involved removing the surface soil with the assistance of a tractor and manually collecting the *P*. *polyphylla* larvae. To ensure cleanliness, the larvae underwent an extensive washing procedure involving five cycles with a 0.05% chlorine solution. Afterward, each larva was placed in a small plastic container containing damp peat moss (70%), previously sterilized through autoclaving. In the laboratory, these containers were positioned in a dark room at room temperature, initiating a quarantine phase to monitor larval survival. Those larvae that successfully completed this phase became the control hosts for subsequent host preference experiments. Conversely, insect larvae succumbing to *R*. *regina* infection were moved to a white trap to gather dauer larvae [25]. The collected nematode larvae then underwent a thorough cleansing process to select the dauers, including a 50-minute soak in a 0.1% Sodium Dodecyl Sulfate (SDS) solution, followed by five successive rinses with sterile distilled water, each lasting 30 minutes [26]. Subsequently, the surviving dauer larvae were used in three distinct experiments: host preference trials, energy reserve analysis, and *R*. *regina* experimental evolution. Simultaneously, we harvested wild nematodes from the deceased *P*. *polyphylla* larvae to assess the life history traits of interest. These wild nematodes served as the control group in the analysis of life history traits.

#### Spodoptera frugiperda

The pupae of *S*. *frugiperda* were collected from corn fields in Tarimbaro, Michoacan, Mexico (19°46’19.0"N 101°08’51.6"W) and were breed in an insectary at UNAM. The Spodoptera larvae were breed on a diet comprising wheat bran (150 g), maize powder (100 g), yeast powder (30 g), agar (20 g), ascorbic acid (3 ml), vitamins (0.1 g), formaldehyde (2 ml), glacial acetic acid (4 ml), and distilled water (1500 ml) [27, 28]. These larvae were housed in plastic containers within an incubator under dark conditions, maintaining a temperature of 26°C and a humidity level of 70%. Pupae were collected for use in host preference experiments (as the experimental host) and in *R*. *regina* experimental evolution to establish the alternate host line. Before initiating the experiments, all pupae underwent a cleansing procedure, involving treatment with a 0.05% chlorine solution, followed by thorough rinsing with sterile distilled water.

#### Tenebrio molitor

*T*. *molitor* adults were obtained from an Insectary at UNAM. The insects were kept under controlled conditions, maintained at a temperature of 27°C in complete darkness. The mealworms were nourished with sterilized wheat bran and corn flour as their primary food sources, supplemented with apple slices provided every other day. All food items were subjected to sterilization (subjected to 125°C±2°C for 15 minutes) to prevent any infections [29]. Adult beetles were sacrificed by placing them in a -20°C freezer for 30 minutes. Subsequently, they underwent a meticulous cleaning process involving a 0.05% chlorine solution, followed by thorough rinsing with sterile distilled water. These deceased *T*. *molitor* were utilized in the host preference experiments as experimental hosts and, in the *R*. *regina* experimental evolution experiments to establish the saprophytic line.

### Host preference

**Fig 1:**
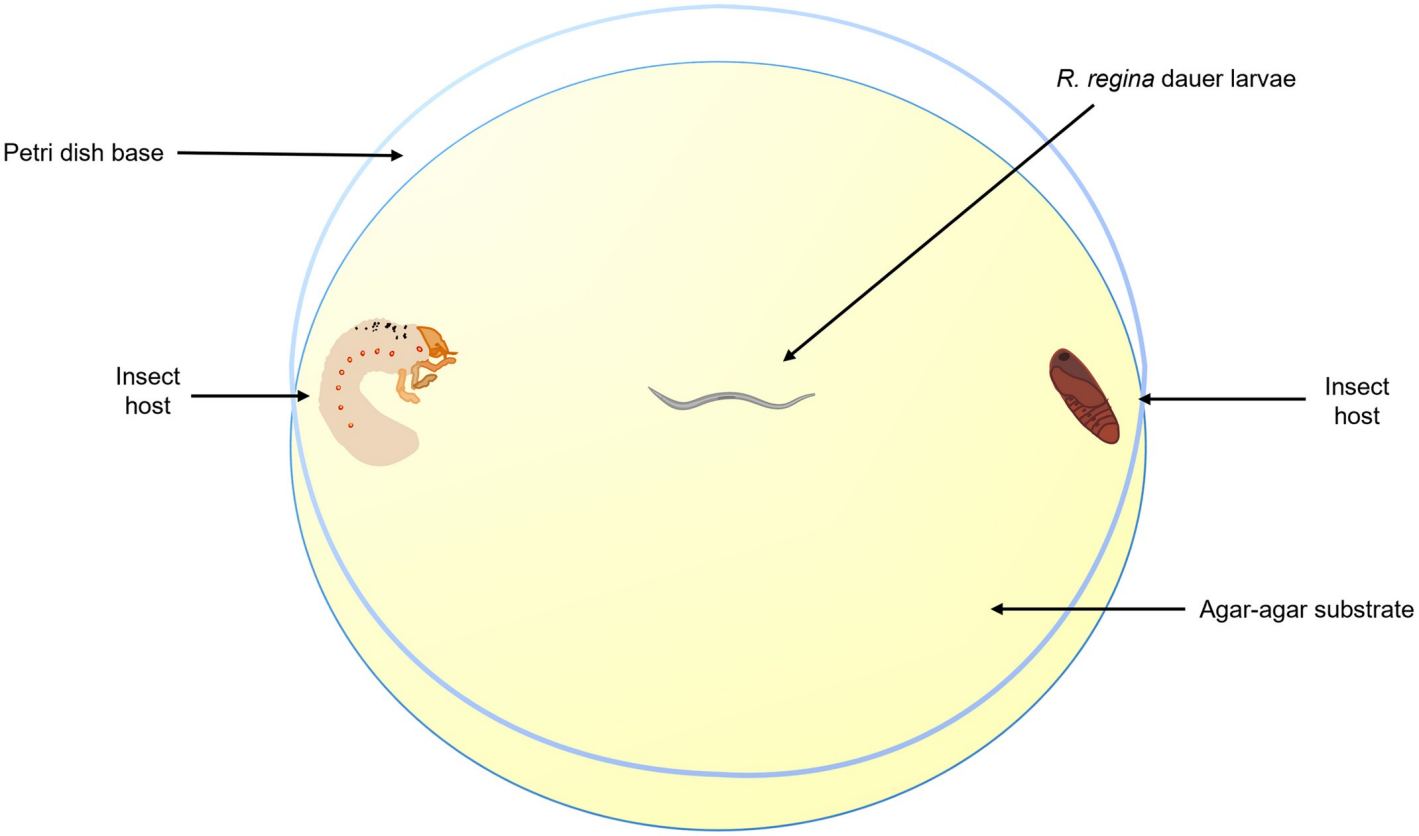
Conductual chambers to test *R*. *regina*’s host preference. The arena was established in a Petri dishes (150 x 20 mm) filled with 100 ml of Agar-agar (1.5%) as substrate. Positioned in the center we placed one dauer larvae, while at both ends, two different hosts combinations (*P*. *polyphylla* vs *S*. *frugiperda*; *P*. *polyphylla* vs *T*. *molitor*; *S*. *frugiperda* vs *T*. *molitor*). The hosts were introduced 15 minutes before the dauer larvae to allow for proper acclimatization.

To evaluate nematode food preferences, we conducted experiments involving different pairs of hosts: *P*. *polyphylla vs*. *S*. *frugiperda*, *P*. *polyphylla vs*. *T*. *molitor*, *and S*. *frugiperda vs*. *T*. *molitor*. These experiments were carried out in large Petri dishes measuring 150 x 20 mm, with a substrate of 1.5% agar-agar. Within each dish, two distinct hosts were positioned at opposite ends, and a single *R*. *regina* larva was centrally placed using a micropipette with 2 μL of PBS (Phosphate-buffered saline) as a vehicle (Fig 1). To record the foraging behavior of the dauer larvae, we utilized a stereomicroscope (Leica EZ4) and implemented the following observation method: We divided the experiment into three intervals, spanning a total of 18 hours. The first interval involved five minutes of observations every half-hour, resulting in a total of 12 observations within the initial six hours. The second interval consisted of five minutes of observations every hour, yielding a total of six observations in the subsequent six hours. The third interval included five minutes of observations every two hours, totaling three observations in the final six hours. Throughout the observation period, we documented the trajectory of the larvae and noted instances of proximity to a host. Host preference was recorded if the larva made physical contact with the host and remained in proximity until the end of the experiment. We conducted three replicates of 30 trials (n = 30) for each host combination. The observation schedule was determined through trial experiments, revealing heightened dauer larval activity during the initial 12 hours. Within this timeframe, some larvae established contact with their chosen host, while others remained in proximity (1.5 centimeters). The final six-hour phase was employed to assess whether the dauer larvae-maintained contact with the selected host or moved away from it.

### Experimental evolution

We used *R*. *regina* for conducting experimental evolution [30, 31]. In this method, we systematically manipulated the environmental conditions provided by the insect host within a controlled laboratory setting. This allowed us to closely examine the ensuing evolutionary changes in nematodes over a span of 50 or 53 generations, depending on the host. Such an approach serves as a robust means to explore how fitness varies in response to distinct environments, with our focus specifically directed towards the involved hosts. Through this process, we established two distinct evolution lines: one utilizing *S*. *frugiperda* pupae (referred to as the Alternate host line), and the other involving *T*. *molitor* carcasses (referred to as the Saprophytic line). These experiments were conducted within culture media placed in Petri dishes measuring 150 x 20 mm, with a substrate of 1.5% agar-agar. For both host types, eight insects were utilized in each culture medium. Both the nematodes and insects underwent the washing process as detailed in the preceding sections, and the culture media were prepared under aseptic conditions. After 8–10 days, the culture media were rinsed with sterile distilled water using a Pasteur pipette, enabling the collection of nematodes and their transfer to fresh mediums with the corresponding host. These cultures were maintained under aseptic conditions within an incubator, ensuring complete darkness, a constant temperature of 26°C, and a relative humidity level of 70%. These procedures were repeated during each passage. The evolution lines were maintained for a period of five months before conducting the analysis of life history traits. The number of generations were 50 for *S*. *frugiperda* pupae and 53 for *T*. *molitor*. This difference in number of generations is attributed to the smaller size of the *S*. *frugiperda* colony, resulting in fewer insects obtained per month for the lepidopteran host. Each treatment has 4–5 independent lines of breeding and, as no differences were found between breeding lines for *S*. *frugiperda* or *T*. *molitor*, data were pooled and reported as a single line.

### Life history traits analysis

#### Female and male lengths

Nematodes were collected from the deceased *P*. *polyphylla* larvae and the culture media of each evolution line, employing delicate brushes and a stereomicroscope (Leica EZ4). Subsequently, they were individually placed on a small Petri dish base, and heated formaldehyde (4%) was introduced to facilitate elongation and measurements. Based on distinctive morphological features, we meticulously separated adults from the larvae, collecting 50 females and 50 males per evolution line. Following this, a droplet of an ethanol-glycerin solution (50:50 v/v) was applied to a slide to carefully mount the nematodes, after which a coverslip was added. The specimens were observed under an optical microscope (ZEISS, Axio Imager 2), and measurements were obtained using the ZEN Digital Imaging for Light Microscopy software (RRID:SCR_013672).

#### Survival and larvae production

The outlined procedure aimed to assess nematode survival and larvae production per adult nematode pair from each host line. Initially, gravid females (n = 30) were individually collected from each evolution line and placed in culture media to monitor larvae emergence. The culture media were set up in small Petri dishes, featuring a substrate of agar-agar (1.5%) and a small piece of raw meat (0.005 g) provided as ad libitum food. These dishes were maintained in an incubator under conditions of darkness, at a temperature of 26°C, and a relative humidity of 70%. Every 24 hours, gravid females were transferred to new fresh mediums, with simultaneous observation for larvae emergence. Once larvae emerged, they were collected, and their survival was tracked. Developmental rate was recorded at 24-hour intervals until they reached the adult stage. Subsequently, two young adults (male and female) from different mothers were paired and relocated to new fresh mediums (n = 30 adult pairs/evolution line). Daily transfers to new fresh cultures were conducted, and the presence of larvae was verified. This procedure was repeated until the adults died, ceasing the production of larvae.

#### Dauer larvae’s energy reserves

Freshly emerged dauer larvae were harvested from the deceased *P*. *polyphylla* larvae and the culture media of each evolution line. These larvae were placed into conical test tubes kept at a cold temperature, containing an SDS solution (0.1%) for 50 minutes to facilitate thorough cleansing. Following this, the concentrated dauer larvae pellets underwent five rinses with sterile distilled water and were then transferred to Eppendorf tubes for precise weighing using an analytical scale. Energy reserves were measured with the kits Trygliceride Quantification kit (Sigma ©) and Glycogen Assay kit (Sigma ©) according to the manufacturer’s instructions. For triglyceride analysis, 100 mg samples (n = 10/evolution line) of dauer larvae were used, while for glycogen analysis, samples of 10 mg (n = 10/evolution line) were utilized.

### Statistical analysis

Host preference was registered as the frequencies of dauer larvae choice among each host combination (i. e. *P*. *pollyphylla* vs *S*. *frugiperda*; *P*. *polyphylla* vs *T*. *molitor*; *S frugiperda* vs *T*. *molitor*). Then, the data were analyzed by a 2x2 contingency table, with the chi-squared (χ^2^) test being applied to assess interrelationships between each host combination. Life history traits data (adult size, larval production, and triglyceride and glycogen content) were subjected to normality and homoscedasticity assumptions through the Shapiro-Wilk test and Fligner-Killeen test, respectively. Data conforming to these assumptions underwent analysis using ANOVA test, with Tukey post-hoc test used to detect significant differences between groups. Those that did not meet these assumptions were analyzed by Kruskal-Wallis test, with Bonferroni post-hoc test to test differences among groups. Survival was analyzed using Log-Rank (Mantel-Cox) with multiple pairwise comparisons. All statistical analyses were performed using IBM SPSS Statistics 22 software.

## Results

### Host preference

**Fig 2:**
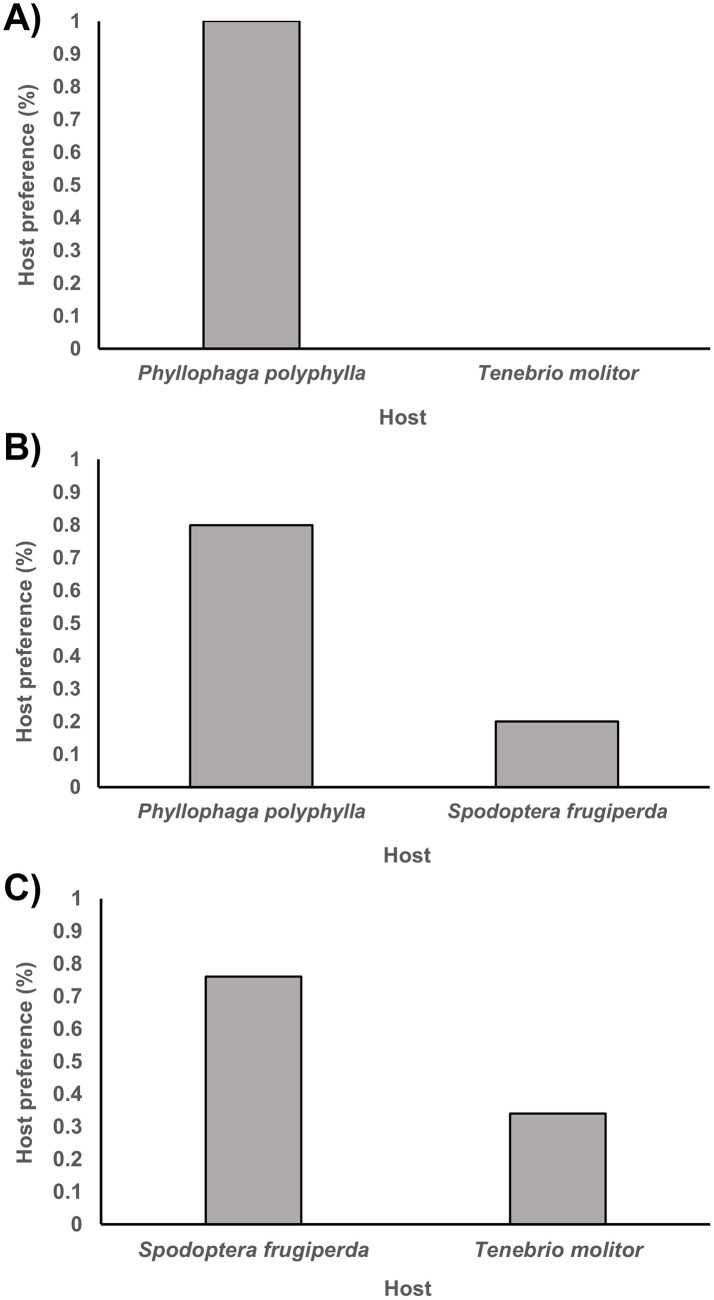
Frequency of *R*. *regina* host preference by host pair combination. Hosts are depicted on the X-axis, with the Y-axis indicating the preference frequency percentage exhibited by dauer larvae. A) *P*. *polyphylla* vs. *T*. *molitor*; B) *P*. *polyphylla* vs. *S*. *frugiperda*; C) *S*. *frugiperda* vs. *T*. *molitor*.

We first tested *R*. *regina* host preference among different pair hosts combinations to know its foraging behaviour according to host availability. Control host *P*. *polyphylla* was preferred with a 100% frequency over the experimental host *T*. *molitor* (Chi squared test χ^2^ = 72.00, df = 1, p < 0.001; Fig 2a). We also saw a notable 80% frequency of preference for the Control host over the experimental host *S*. *frugiperda* (Chi squared test χ^2^ = 32.26, df = 1, p < 0.001; Fig 2b). Lastly, when testing the preference between both experimental hosts, *R*. *regina* preferred *S*. *frugiperda* with a 70% frequency over *T*. *molitor* (Chi squared test χ^2^ = 32.00, df = 1, p < 0.001; Fig 2c). The results showed that *R*. *regina* tend towards a parasitic lifestyle, exploiting alive insects, rather than a saprophytic lifestyle, exploiting dead insects. Also, the results suggest a host specificity for *P*. *polyphylla* over an alternate host as *S*. *frugiperda*. The specificity in host preference, demonstrate that host choice is not arbitrary and suggests mechanisms of recognizing and location of the preferred host, which means a close evolutionary bound.

### Life history traits

#### Adult sizes

**Fig 3:**
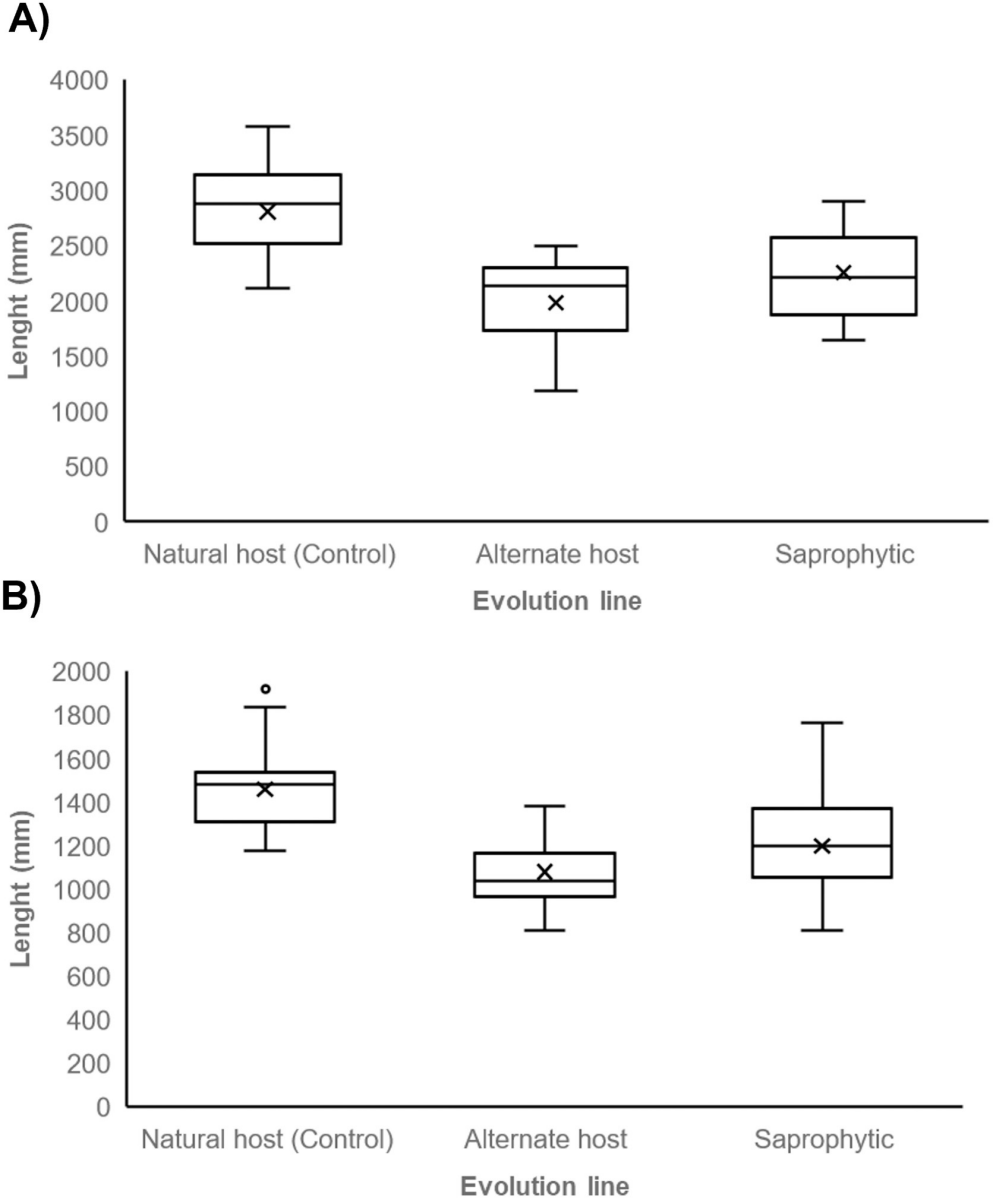
*R*. *regina* adult size. Average length (± S.E.) of females (A) and males (B) nematodes from the natural host, alternate host, and saprophytic lines, is depicted. Different letters mean significant differences (p < 0.01).

We found that adult nematodes from the Control line exhibited greater size, compared to those from both experimental lines (alternate host line and saprophytic line) (Table 1; Fig 3). We detected significant variations in female size among the control and experimental lines (Kruskall-Wallis test χ^2^ = 51.319, df = 2, p < 0.05; Fig 3a). Specifically, these disparities were pronounced between the Control line females and those of each experimental line (post hoc Bonferroni test: Control line vs alternate host line p < 0.01; Control line vs saprophytic line p < 0.01). However, no distinctions were observed between females’ size from the alternate host and saprophytic lines (post hoc Bonferroni test p = 0.092). Moreover, we noted significant discrepancies in the males’ size across the investigated lines (Kruskall-Wallis test χ^2^ = 41.955, df = 2, p < 0.01; Fig 3b). The Control line males had a greater size compared with the alternate host (Bonferroni post hoc test p < 0.01), and the saprophytic line males (Bonferroni post hoc test p < 0.01). Nonetheless, there were no distinctions between the latter two (Bonferroni post hoc test p = 0.017). As predicted, *R*. *regina* body size is favored in its natural host, while the establishment and development in the alternate host and saprophytic environment negatively modified this trait. The observed patterns suggest that *R*. *regina* exploits and copes efficiently the environment within its natural host, and that these capabilities are limited when establishing in an alternate host or a saprophytic habitat.

**Table 1:** *R*. *regina* life history’s strategies. The average and standard error of each life history trait of *R*. *regina*, measured within the hosts *P*. *polyphylla*, *S*. *frugiperda*, and *T*. *molitor*, are displayed (F) = Females, (M) Males.

	*P*. *polyphylla* (Natural host)	*S*. *frugiperda* (Alternate host)	*T*. *molitor* (Saprophyte habitat)
**Adult size (mm)**	2.8 ± 44.21 (F) 1.4 ± 28.36 (M)	1.9 ± 56.6 (F) 1.0 ± 24.35 (M)	2.2 ± 48.7 (F) 1.7 ± 35.98 (M)
**Longevity (days)**	7.1 ± 0.4 (F) 7.4 ± 0.4 (M)	5.0 ± 24.3 (F) 4.1 ± 0.37 (M)	3.7 ± 0.43 (F) 3.1 ± 0.3 (M)
**Larvae production/adult pair**	151 ± 40.3	26.1 ± 14.2	7.5 ± 6.2
**Glycogen (μg/mg)**	0.13 ± 0.03	0.10 ± 0.04	0.05 ± 0.02
**Triglycerides (nmol/mg)**	14.08 ± 1.43	11.03 ± 3.47	16.48 ± 3.30

#### Survival

**Fig 4:**
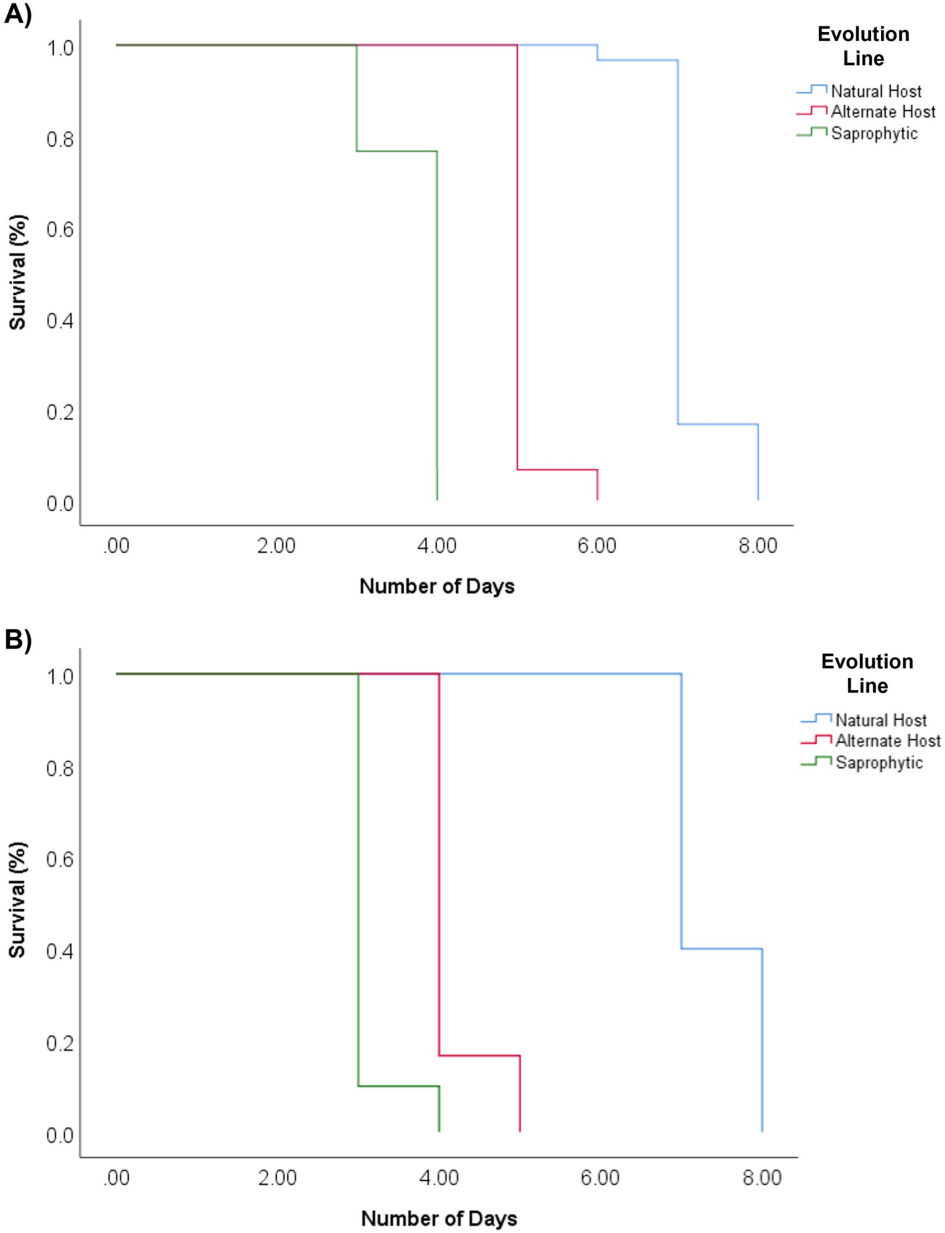
*R*. *regina* female and male survival. Kaplan Meier plots show *R*. *regina* females (A) and males (B) nematodes survivals, derived from each evolution line: natural host (blue), alternate host (red) and saprophytic (green).

The survival was measured by the elapsed days since nematodes’ birth until death. We observed significant variation among females’ survival from the studied lines (Log Rank test χ^2^ = 124.93, df = 2, p < 0.001; Table 1; Fig 4a). Control line females exhibited longer survival than those from the alternate host (Log Rank test χ^2^ = 54.47, df = 1, p < 0.001) and saprophytic lines (Log Rank test χ^2^ = 56.47, df = 1, p < 0.001). Additionally, alternate host line females lived longer than those from saprophytic line (Log Rank test χ^2^ = 56.47, df = 1, p < 0.001), the latter with the shortest survival. Similarly, we observed significant differences in males survival across the Control and experimental lines (Log Rank test χ^2^ = 115.53, df = 2, p < 0.001; Table 1; Fig 4b). Control line males exhibited longer survival than those from alternate host (Log Rank test χ^2^ = 66.33, df = 1, p < 0.001) and saprophytic lines (Log Rank test χ^2^ = 65.7, df = 1, p < 0.001). Furthermore, males from the alternate host line lived longer than those from the saprophytic line (Log Rank test χ^2^ = 47.07, df = 1, p < 0.001). Consistent with the observed pattern in females, saprophytic line males demonstrated the shortest survival among the three studied lines. These results correspond with our predictions that the natural host favors this trait over the experimental hosts. Similar to adult body size, modifications in nematode survival demonstrate the effect of particular selective pressures in the alternate host and saprophytic environment.

#### Larvae production

**Fig 5:**
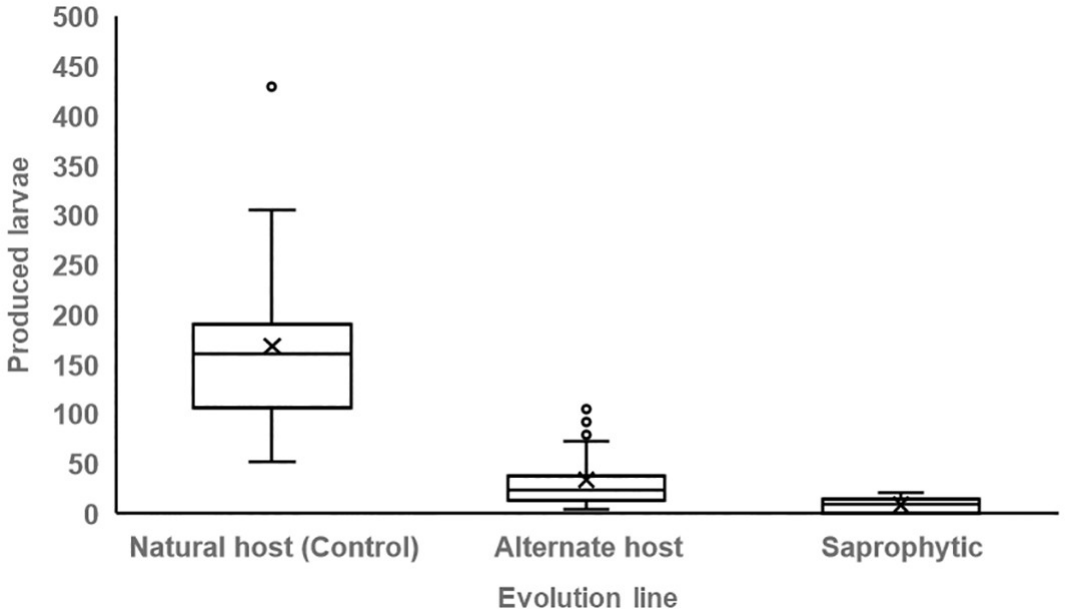
*R*. *regina* larvae production. Average number of larvae (± S.E.) engendered by pairs of adults derived from the natural host, alternate host and saprophytic lines is displayed. Different letters mean significant differences (p < 0.01).

We quantified larvae production per adults pair to reveal the nematode’s reproductive efficiency. We found that the larvae production was significantly different among the three lines (Kruskall Wallis test χ^2^ = 65.079, df = 2, p < 0.05; Table 1; Fig 5). Control line adults exhibited superior reproductive performance by producing a significantly greater number of larvae compared to the alternate host (post hoc Bonferroni test p < 0.01) and the saprophytic lines (post hoc Bonferroni test p < 0.01). Differences were also evident among the experimental lines (post hoc Bonferroni test p < 0.01), with the saprophytic nematodes displaying the lowest reproductive performance. Our predictions coincide with the results, as the reproductive output of the Control line is significantly higher than in the experimental lines. According to theory, the reproductive output is related with adult’s body size and survival. The results were consistent with this notion, as the reproductive efficiency corresponds with the previous life history traits expressed in each evolution line.

#### Energy reserves content (glycogen and triglycerides)

**Fig 6:**
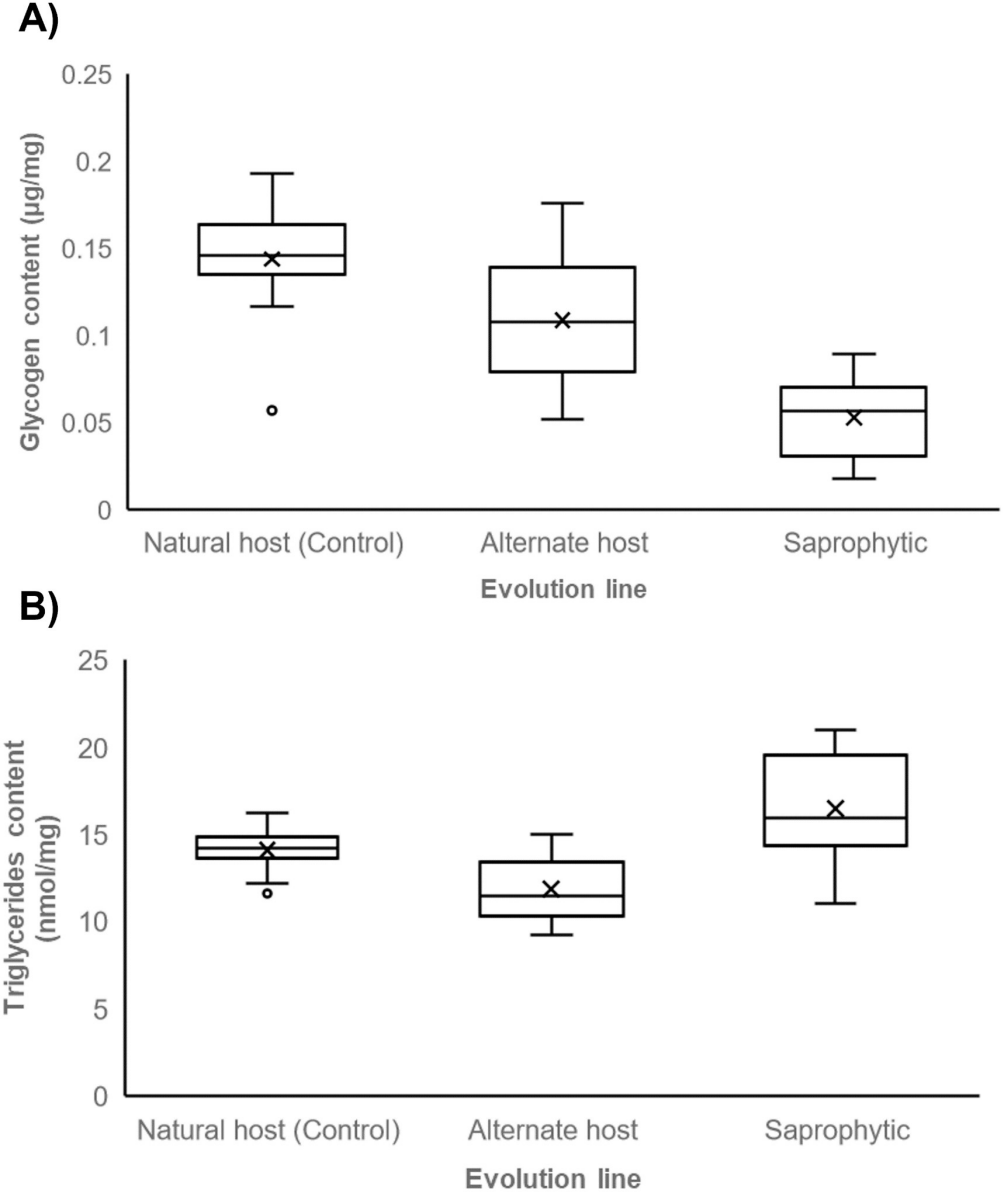
Dauer larvae energy reserves. Average glycogen (μg/mg) (A) and triglycerides (nmol/mg) (B) content in dauer larvae derived from *P*. *polyphylla*, *S*. *frugiperda* and *T*. *molitor*. Different letters mean significant differences (p < 0.01).

Glycogen and triglycerides content are important life history traits since them correlates with dauer larvae dispersal and survival. Thus, quantifying these molecules may reveal dauer larvae physiological quality. We found that glycogen content differed significantly among the Control and experimental groups (Kruskall Wallis χ^2^ = 15.185, df = 2, p < 0.01; Table 1; Fig 6a). Control line dauer larvae showed similar glycogen levels to those from alternate host line (post hoc Bonferroni test p = 0.211), but significant differences were detected when compared to saprophytic line (post hoc Bonferroni test p < 0.01), which displayed notably reduced levels. However, no significant differences in glycogen content were observed between both experimental lines (post hoc Bonferroni test p = 0.025). Furthermore, the triglyceride content also exhibited significant differences (χ^2^ = 9.725, df = 2, p < 0.01; Table 1; Fig 6b). Specifically, Control line triglyceride levels did not significantly differ from those in alternate host (post hoc Lincoln test p = 0.09) or saprophytic lines (post hoc Lincoln test p = 0.311). However, notable distinctions were observed between the latter two, with significantly higher triglyceride levels in saprophytic line (post hoc Lincoln test p < 0.01). Our predictions did not match with the obtained results. We expected higher glycogen levels only in Control line, but we found a similar content among Control and Alternate host lines. This also was expected for triglycerides, however, we found similar content between Control and both experimental lines, with Saprophytic line having higher values than Alternate host line.

## Discussion

Our study shows a skewed preference of *R*. *regina* for *P*. *polyphylla*, aligning with the benefits it provides for the nematode’s growth, survival, and reproduction. This preference sharply contrasts with the less favored hosts, *S*. *frugiperda* and dead *T*. *molitor*. This investigation marks the pioneering exploration of foraging optimization in a facultative parasitic nematode, elucidating its host preference and the evolution of life history traits. Despite the ecological and evolutionary significance of facultative parasites, research on their food preferences remains limited [5–9, 32]. By examining the evolution of parasitism in facultative parasites through the lens of optimal foraging, we can uncover the underlying basis of host preference and its evolutionary implications [33, 34]. In this context, our study offers insights into the foraging behavior of *R*. *regina*. The preference for living insects and the associated benefits support the idea of a skew towards parasitism [1, 35]. The transition to parasitism in nematodes occurs when they develop strategies for locating hosts, and the fitness gained from parasitism surpasses that of a free-living existence [34, 36–38]. While previous studies often focused solely on reproductive efficiency, our analysis of life history traits illustrates that entomopathogeny is more advantageous for *R*. *regina* than a saprophytic lifestyle [38]. However, the performance of life history traits varies between *P*. *polyphylla* and *S*. *frugiperda* hosts, suggesting that *R*. *regina* cannot exploit these hosts equally [39, 40]. Even when there may be a fitness disadvantage, using live hosts incurs fewer costs than using dead hosts. For instance, employing living hosts results in higher larvae production in *S*. *affine* and *S*. *kraussei* compared to using dead hosts [41]. These findings underscore how different host species can modify the life history traits of nematodes, as observed in the length of males of *Aplectana hylambatis*, which significantly varies among four anuran host species, or *Nippostrongylus brasiliensis*, which undergoes changes in development, lifespan, and egg production when using an alternate rodent host [42, 43]. Collectively, our findings suggest that entomopathogenic nematodes exhibit optimal decision-making when selecting and utilizing hosts.

The preference of *R*. *regina* for *P*. *polyphylla* suggests the involvement of a specific recognition mechanism, akin to the insect recognition strategies observed in EPN [18, 44–46]. Foraging behavior in EPN is typically triggered by the detection of CO_2_, pheromones, or other volatile compounds emitted by insects [16, 47, 48]. However, this attraction is often specific, as some nematodes exhibit greater attraction to certain insect odors over others [17]. For example, in *S*. *scapterisci*, a natural entomopathogen of crickets, which displays a stronger attraction to the odorant 3-hydroxy-2-butanone emitted by crickets compared to odors from other insects [16]. A similar phenomenon likely occurs in *R*. *regina*, where the odor of *P*. *polyphylla* is more attractive than that of *S*. *frugiperda* or *T*. *molitor*. Consequently, host preference appears to result from specific recognition mechanisms driven by the maximization of fitness [9, 49]. This notion is supported by the benefits that *R*. *regina* gains from exploiting *P*. *polyphylla*. The life history traits of nematodes significantly impact their ecology and evolution [50–53]. For instance, long-lived nematodes tend to achieve larger sizes and extended reproductive periods, especially in iteroparous species. Larger adult sizes are associated with increased reproductive capacity, such as higher egg production [54, 55]. Additionally, larger adults have greater access to food, occupy better places to live as parasites, and have more reproductive opportunities compared to smaller ones [53]. In summary, higher values in life history traits translate into short- and long-term advantages, thereby reinforcing host preference. Taking a life history approach, the benefits derived from using *P*. *polyphylla* suggest that *R*. *regina* is better adapted to this host [56–58]. Attributes such as virulence and immune evasion of *R*. *regina* may contribute to the efficient exploitation of *P*. *polyphylla* [59]. The costs associated with using alternate hosts or environments underscore the susceptibility of *R*. *regina* to habitat shifts [60]. Resource quantity and quality, along with the acquisition of resources by the nematode, may be key limiting factors [40, 58, 61, 62]. Research focused on measuring the nutritional input of insect hosts can help explain this phenomenon [61]. Additionally, the concept of the threshold elemental ratio (TER), a well-established tool providing a meaningful index of elemental imbalance between a consumer and its food resource, can be explored to unveil the connection between the physiological attributes of nematodes and the food quantity in the environment, shedding light on the observed life history patterns [63, 64].

While *R*. *regina* is a natural entomopathogen of *P*. *polyphylla*, it remains uncertain whether this specificity extends to other white grubs [65]. White grubs exhibit a diverse array of behavioral, morphological, physiological, and immunological defenses, suggesting the possibility of different host preferences [66, 67]. Therefore, future research should explore how these attributes influence the foraging behavior of nematodes. Further investigation into the energetic costs and benefits, as well as the time investment in foraging, can help ascertain the degree of specificity and validate the concept of optimization [3, 8, 68]. Understanding the intricacies of these factors will provide valuable insights into the mechanisms underlying host preference in *R*. *regina* and other entomopathogenic nematodes. Another crucial aspect to consider is field preference, which could potentially be influenced by host density and competition [9, 24]. Examining the behavior of *R*. *regina* in natural environments will contribute to a more comprehensive understanding of its host preference dynamics. All these perspectives may shed light on whether preference for alternative hosts or saprophytic environments is context-dependent or if the preference for the natural host, *P*. *polyphylla*, predominates. This approach will contribute to a more nuanced comprehension of the host preference behavior of *R*. *regina* and its implications in diverse ecological settings.

As predicted, the unavailability of *P*. *polyphylla* leads to a shift in host preference towards *S*. *frugiperda* rather than *T*. *molitor* carcasses. This shift in preference may be attributed to the hypothesis that foraging behavior is primarily driven by the CO_2_ emissions of living hosts, recognized as a universal molecule for host detection [69–71]. Undoubtedly, *S*. *frugiperda* emits odors that differ from those of the natural host. However, when mixed with CO_2_, they signal the potential presence of a host, making the nematode more attracted to the lepidopteran host over the deceased coleopteran. After host recognition, nematodes encounter hostile environments, such as immune responses and variations in resource quantity and quality [34, 72, 73]. The successful establishment and survival of *R*. *regina* in *S*. *frugiperda* may be considered an exaptation resulting from its parasitic life in *P*. *polyphylla* and maybe, white grubs, in general. However, the nematode’s life history traits have been significantly reduced in the lepidopteran host, potentially due to the exploitation of a smaller host, as indicated by a study on *H*. *megidis* where the size of dauer larvae and larvae production increased with the size of the host [74]. Another possible factor could be the host’s immune response, because is a significant selective pressure modifying the life history of parasites [73, 75–78]. Previous research has confirmed that *S*. *frugiperda* exhibits a robust immune response against EPN [79]. Therefore, comparing the immune response of *S*. *frugiperda* versus *P*. *polyphylla* may reveal whether *R*. *regina* incurs more costs combating the immune response of the former. Immune defense can compromise growth and reproduction due to the allocation of resources necessary for survival [53, 56, 80]. The trade-off between investment in immune response versus other life-history traits has been studied in vertebrate parasitic nematodes [73, 76] but remains to be tested in EPN. Therefore, it is imperative to conduct studies deciphering the molecular dialogue between host immune effectors and the nematode’s insulin-like signaling cascade, ultimately influencing phenotypic expression. The insulin-like signaling pathway stands out because it directly influences the nematode’s life history traits, with numerous genes implicated in regulating metabolism, immunity, and physiology [81, 82]. Studies are required to explore the intricate molecular network between host immune effectors and the nematode’s insulin-like signaling cascade, ultimately influencing phenotypic expression.

The limited preference for dead *T*. *molitor* and the associated costs in *R*. *regina’s* life history underscore the inadequacy of saprophagy as a strategy. Phylogenetic and empirical studies suggest that saprophagy precedes entomopathogeny in the evolutionary history of nematodes [35, 83–85]. Transitioning to a new lifestyle, such as entomopathogeny, involves substantial modifications in behavior and fitness [13, 85]. This is supported by the observation that species of the Steinernema genus exhibit avoidance behaviors when it comes to dead insects [86]. Although species of Steinernema and Heterorhabditis can exploit dead insects, their reproductive efficiency is significantly reduced [41, 87]. In the case of *R*. *regina*, the reduced values of life history traits, even lower than in *S*. *frugiperda*, emphasize that saprophagy is costly for this nematode. Smaller and shorter-lived adults result in lower larvae production and fewer mating events, reducing fitness [34, 52, 56, 88]. While suboptimal phenotypes pose evolutionary disadvantages, they might be better adapted to immediate ecological shifts compared to parasitic phenotypes, particularly in situations where suitable hosts become scarce [10]. This underscores the dynamic nature of nematode strategies and their ability to adapt to changing ecological conditions.

The triglyceride content between *P*. *polyphylla* and dead tenebrios was not statistically significant. According to life history theory, resource-limited habitats promote the optimization of resource allocation to maximize fitness [57, 81]. In this case, the reduction in larvae production is compensated by heightened triglyceride reserves production. These reserves are crucial for dauer larvae survival, serving as the energetic currency for the aerobic metabolic pathway [89–91]. Given the free-living nature of the dauer stage, elevated triglyceride reserves may extend survival in the soil, increasing the chances of encountering suitable hosts or habitats. Conversely, glycogen values were the lowest in dead tenebrios. The availability of this molecule is pivotal, as it is required for anaerobic metabolism during the parasitic stage of EPN [90]. This cost is particularly relevant for *R*. *regina*, as low glycogen levels are associated with reduced infectivity [92, 93]. With low glycogen levels, *R*. *regina* may be less successful in infecting living hosts after growing in a saprophytic environment. The differences between glycogen and triglyceride reserves may be attributed to the availability of these molecules in the *T*. *molitor* carcass. Studies demonstrate that the sugar and fat reserves of *T*. *molitor* are utilized during pupal-to-adult metamorphosis, with sugars serving as the primary energy source [94, 95]. Consequently, the limited availability of sugars in *T*. *molitor* is reflected in the low levels of glycogen stored in the dauer larvae.

The saprophytic phenotype in *R*. *regina* may be attributed to the nutritional value of the carcass. The detection of available resources can lead to adjustments in the life history schedule, a phenomenon known as phenotypic plasticity [40, 96–98]. Therefore, it is plausible that the quality and quantity of resources within the carcass are the determining factors behind the observed differences in life history traits [98]. Given that bacteria serve as the primary food source for a bacteriophage nematode like *R*. *regina*, future research may explore the bacterial biomass present in both the host and the nematode. Characterizing and quantifying fatty acid profiles can provide insights into the bacterial biomass input from the host and the nutritional status of the nematode, as these profiles serve as chemotaxonomic markers for bacteria [99–101]. This approach can help elucidate the differences in life history traits observed among various hosts and environments. Furthermore, the application of metagenomic studies can offer a comprehensive view of the bacterial composition within nematodes, providing insights into their symbiotic stability and metabolic capabilities [19, 102–106]. Given that *R*. *regina* relies on associated bacteria to exploit different hosts, macrobiotic studies can reveal which bacteria are retained or lost and how these changes impact the nematode’s life history. This multi-pronged approach will contribute to a deeper understanding of the intricate interactions between nematodes, their hosts, and associated bacteria, shedding light on the factors influencing their phenotypic expression.

Our findings have important implications for the practical application of EPN in the field of biological control and for evolutionary-ecology research. We propose the following recommendations: (a) Expand the entomopathogenic specie catalog. It is imperative to broaden our comprehension of entomopathogenic lineages beyond the traditionally acknowledged families like Heterorhabditidae and Steinernematidae [12, 13, 107]. Nematode species, such as *R*. *regina*, exhibit entomopathogenic traits akin to established EPN. Investigating their dietary preferences and well-developed life histories can furnish evidence of their entomopathogenic nature. Given the convergent evolution of entomopathogeny, we anticipate that additional nematode lineages may behave as facultative parasite species [35, 38]. This acknowledgment could lead to the identification of more nematode species serving as excellent models for biological control and research across life history, coevolution, ecology, and parasitology; (b) Significance of studying nematode host preference. In the realm of biological control, scrutinizing nematode foraging behavior is pivotal for assessing their effectiveness in locating and infecting target insects, especially when these insects are alternate hosts [108]. Beyond biological control, this analysis can be extended to explore the neurophysiology of host preference [109]. Identifying changes in nematode olfactory circuits can unveil the mechanisms behind optimal foraging, particularly in the context of facultative parasites. Such studies contribute to framing an understanding of the evolution of parasitism within the Nematoda; (c) Significance and consequences of life history evolution. In the field of biological control, it is crucial to acknowledge that rearing nematodes in vivo (using alternate hosts) or in vitro (artificial media) can induce alterations in their life history traits [110]. Nematode rearing within suboptimal hosts or growth media can influence critical ecological and evolutionary factors, potentially constraining the practical implementation of nematodes in pest control [111, 112]. Understanding the breadth of life history traits is essential, as the long-term success of nematodes hinges on them. We advocate for the rearing of naturally associated nematodes with pest insects, ensuring enhanced transmission through optimized foraging behaviors and increased fitness [46, 111]. Furthermore, life history theory lacks a comprehensive framework for facultative parasites [34, 53]. Our study contributes to this field, suggesting that facultative parasites respond to similar selective pressures as strict parasites. However, recognizing the inherent factors shaping the evolution of life history traits in parasites is essential. Molecular or genomic studies could unveil the mechanisms governing the evolution of the life history traits of parasites.

## Conclusions

Facultative parasites play a crucial role in evolutionary ecology, and studying them offers a unique opportunity to unravel the factors influencing the choice between a parasitic and free-living lifestyle. The concept of optimal foraging is central to understanding the evolution of parasitism, particularly the behaviors related to recognition and localization crucial for finding suitable hosts. The strong preference of *R*. *regina* for *P*. *polyphylla* suggests that this choice maximizes fitness compared to using an alternate host or environment. This skew towards a specific host implies a genuine entomopathogenic lifestyle, indicative of a stricter rather than facultative parasitic habit. To delve deeper into this, we recommend exploring the energetic costs and benefits associated with foraging within the framework of optimal foraging theory. Moreover, investigating the co-adaptations between the nematode and its symbiotic bacteria would strengthen our understanding of its entomopathogenic nature. Employing a life history theory approach unveils how the nematode has evolved to exploit resources optimally, expressing optimal strategies when within its suitable host and contrasting traits observed in alternate hosts. We propose a more in-depth exploration of the role of host immune responses and nutritional value in shaping the parasite’s life history. Analyzing the nematode’s microbiome across different hosts is enlightening, contributing valuable insights into resource acquisition and exploitation. These research directions will significantly enhance our understanding of the costs associated with shifts between hosts or habitats. Conducting concurrent studies that consider alternate hosts, the parasite’s foraging preferences, and the associated costs and benefits when the host is or is not suitable will provide key insights into the evolution of entomopathogeny. This approach will contribute to our knowledge of the intricate dynamics governing the interactions between parasites and their hosts.

## Supporting information

S1 File (XLSX)

## References

[1] Luong LT, Mathot KJ. Facultative parasites as evolutionary stepping-stones towards parasitic lifestyles. Biol Lett. 2019;15. doi: 10.1098/rsbl.2019.0058 30991912 PMC6501370

[2] Parker GA, Smith JM. Optimality theory in evolutionary biology. Nature. 1990;348: 27–33.

[3] Macarthur RH, Pianka ER. On Optimal Use of a Patchy Environment. Am Nat. 1966;100: 603–609. Available: https://about.jstor.org/terms

[4] Merritt Emlen J. The Role of Time and Energy in Food Preference. Am Nat. 1966;100: 611–617.

[5] Henry LM, Ma BO, Roitberg BD. Size-mediated adaptive foraging: A host-selection strategy for insect parasitoids. Oecologia. 2009;161: 433–445. doi: 10.1007/s00442-009-1381-2 19504128

[6] Sears BF, Schlunk AD, Rohr JR. Do Parasitic Trematode Cercariae Demonstrate a Preference for Susceptible Host Species? PLoS One. 2012;7. doi: 10.1371/journal.pone.0051012 23272084 PMC3525650

[7] Campbell R, Thiemann TC, Lemenager D, Reisen WK. Host-selection patterns of Culex tarsalis (Diptera: Culicidae) determine the spatial heterogeneity of West Nile Virus enzootic activity in Northern California. J Med Entomol. 2013;50: 1303–1309. doi: 10.1603/me13089 24843936

[8] Hubbard SF, Cook RM. Optimal Foraging by Parasitoid Wasps. Journal of Animal Ecology. 1978;47: 593–604.

[9] Manzoli DE, Saravia-Pietropaolo MJ, Arce SI, Percara A, Antoniazzi LR, Beldomenico PM. Specialist by preference, generalist by need: availability of quality hosts drives parasite choice in a natural multihost–parasite system. Int J Parasitol. 2021;51: 527–534. doi: 10.1016/j.ijpara.2020.12.003 33713648

[10] Johnson PTJ, Calhoun DM, Riepe TB, Koprivnikar J. Chance or choice? Understanding parasite selection and infection in multi-host communities. Int J Parasitol. 2019;49: 407–415. doi: 10.1016/j.ijpara.2018.12.007 30894285 PMC6456413

[11] Campos-Herrera R, Barbercheck M, Hoy CW, Stock SP. Entomopathogenic nematodes as a model system for advancing the frontiers of ecology. J Nematol. 2012;44: 162–176. 23482825 PMC3578465

[12] Adams BJ, Fodor A, Koppenhöfer HS, Stackebrandt E, Patricia Stock S, Klein MG. Biodiversity and systematics of nematode-bacterium entomopathogens. Biological Control. 2006;37: 32–49. doi: 10.1016/j.biocontrol.2005.11.008

[13] Dillman AR, Chaston JM, Adams BJ, Ciche TA, Goodrich-Blair H, Stock SP, et al. An entomopathogenic nematode by any other name. PLoS Pathog. 2012;8. doi: 10.1371/journal.ppat.1002527 22396642 PMC3291613

[14] Stock SP, Blair HG. Entomopathogenic nematodes and their bacterial symbionts: The inside out of a mutualistic association. Symbiosis. 2008;46: 65–75.

[15] Bai X, Adams BJ, Ciche TA, Clifton S, Gaugler R, Kim K suk, et al. A Lover and a Fighter: The Genome Sequence of an Entomopathogenic Nematode Heterorhabditis bacteriophora. PLoS One. 2013;8: 1–13. doi: 10.1371/journal.pone.0069618 23874975 PMC3715494

[16] Hallem EA, Dillman AR, Hong A V., Zhang Y, Yano JM, Demarco SF, et al. A sensory code for host seeking in parasitic nematodes. Current Biology. 2011 Mar. doi: 10.1016/j.cub.2011.01.048 21353558 PMC3152378

[17] Dillman AR, Guillermin ML, Lee JH, Kim B, Sternberg PW, Hallem EA. Olfaction shapes host-parasite interactions in parasitic nematodes. Proc Natl Acad Sci U S A. 2012;109. doi: 10.1073/pnas.1211436109 22851767 PMC3435218

[18] Campbell JF, Lewis EE. Entomopathogenic Nematode Host-search Strategies. The Behavioural Ecology of Parasites. 2002. pp. 13–38.

[19] Jiménez-Cortés JG, Canales-Lazcano J, Lara-Reyes N, Rosenblueth M, Martínez-Romero E, Contreras-Garduño J. Microbiota from Rhabditis regina may alter nematode entomopathogenicity. Parasitol Res. 2016;115: 4153–4165. doi: 10.1007/s00436-016-5190-3 27492201

[20] Park HW, Kim YO, Ha JS, Youn SH, Kim HH, Bilgrami AL, et al. Effects of associated bacteria on the pathogenicity and reproduction of the insect-parasitic nematode Rhabditis blumi (Nematoda: Rhabditida). Can J Microbiol. 2011;57: 750–758. doi: 10.1139/w11-067 21867444

[21] Abebe-Akele F, Tisa LS, Cooper VS, Hatcher PJ, Abebe E, Thomas WK. Genome sequence and comparative analysis of a putative entomopathogenic Serratia isolated from Caenorhabditis briggsae. BMC Genomics. 2015;16. doi: 10.1186/s12864-015-1697-8 26187596 PMC4506600

[22] Torres-Barragan A, Suazo A, Buhler WG, Cardoza YJ. Studies on the entomopathogenicity and bacterial associates of the nematode Oscheius carolinensis. Biological Control. 2011;59: 123–129. doi: 10.1016/j.biocontrol.2011.05.020

[23] Schulte F, Poinar GO. Description of Rhabditis (Rhabditoides) regina n. sp. (Nematoda: Rhabditidae) from the body cavity of beetle larvae in Guatemala. Revue de Nematologie. 1991;14: 151–156.

[24] Crossan J, Paterson S, Fenton A. Host availability and the evolution of parasite life-history strategies. Evolution (N Y). 2007;61: 675–684. doi: 10.1111/j.1558-5646.2007.00057.x 17348930

[25] White GF. A Method for Obtaining Infective Nematode Larvae from Cultures. Science (1979). 1927;66: 302–303. doi: 10.1126/science.66.1709.302-a 17749713

[26] Karp X. Working with dauer larvae. WormBook. 2018; 1–19. doi: 10.1895/wormbook.1.180.1 27417559 PMC5237411

[27] Pinto JRL, Torres AF, Truzi CC, Vieira NF, Vacari AM, De Bortoli SA. Artificial Corn-Based Diet for Rearing Spodoptera frugiperda (Lepidoptera: Noctuidae). Journal of Insect Science. 2019;19. doi: 10.1093/jisesa/iez052 31260529 PMC6601867

[28] Ge S, Chu B, He W, Jiang S, Lv C, Gao L, et al. Wheat-Bran-Based Artificial Diet for Mass Culturing of the Fall Armyworm, Spodoptera frugiperda Smith (Lepidoptera: Noctuidae). Insects. 2022;13. doi: 10.3390/insects13121177 36555087 PMC9788468

[29] Punzo F, Mutchmor JA. Effects of Temperature, Relative Humidity and Period of Exposure on the Survival Capacity of Tenebrio molitor (Coleoptera: Tenebrionidae). Journal of the Kansas Entomological Society. 1980;53: 260–270. Available: https://about.jstor.org/terms

[30] Ebert D. Experimental Evolution of Parasites. Evolution. 1998;282. Available: http://science.sciencemag.org/ doi: 10.1126/science.282.5393.1432 9822369

[31] Collins S. Many Possible Worlds: Expanding the Ecological Scenarios in Experimental Evolution. Evol Biol. 2011;38: 3–14. doi: 10.1007/s11692-010-9106-3

[32] Combes C. Ethological Aspects of Parasite Transmission. Am Nat. 1991;138: 866–880.

[33] Parker GA, Stuart RA. Animal Behavior as a Strategy Optimizer: Evolution of Resource Assessment Strategies and Optimal Emigration Thresholds. Am Nat. 1976;110: 1055–1076. Available: https://www.jstor.org/stable/2460030

[34] Poulin R. Evolutionary Ecology of Parasites. Princetown University Press; 2007.

[35] Sudhaus W. Evolution of insect parasitism in rhabditid and diplogastrid nematodes. In: Makarov SE, Dimitrihevic RN, editors. Advances in Arachnology and Developmental Biology. 2008. pp. 143–161. Available: https://www.researchgate.net/publication/288520551

[36] Crook M. The dauer hypothesis and the evolution of parasitism: 20years on and still going strong. International Journal for Parasitology. 2014. pp. 1–8. doi: 10.1016/j.ijpara.2013.08.004 24095839 PMC3947200

[37] Schmid-Hempel P. Evolutionary Parasitology. The Integrated Study of Infections, Immunology, Ecology, and Genetics. Second. Oxford University Press; 2021.

[38] Warburton EM, Zelmer DA. Prerequisites for parasitism in rhabditid nematodes. Journal of Parasitology. 2010;96: 89–94. doi: 10.1645/GE-2277.1 19803545

[39] Lootvoet A, Blanchet S, Gevrey M, Buisson L, Tudesque L, Loot G. Patterns and processes of alternative host use in a generalist parasite: Insights from a natural host-parasite interaction. Funct Ecol. 2013;27: 1403–1414. doi: 10.1111/1365-2435.12140

[40] Araujo SBL, Braga MP, Brooks DR, Agosta SJ, Hoberg EP, Von Hartenthal FW, et al. Undestanding host-switching by ecological fitting. PLoS One. 2015;10. doi: 10.1371/journal.pone.0139225 26431199 PMC4592216

[41] San-Blas E, Gowen SR. Facultative scavenging as a survival strategy of entomopathogenic nematodes. Int J Parasitol. 2008;38: 85–91. doi: 10.1016/j.ijpara.2007.06.003 17662985

[42] Lichtenfels JR. Changes in the Phenotype of the Rat Nematode, Nippostrongylus brasiliensis, after One and 150 Nematode Generations in Hamsters. J Parasitol. 1971;57: 517–525. 5090959

[43] González CE, Gómez VI, Hamann MI. Morphological variation of Aplectana hylambatis (Nematoda: Cosmocercidae) from different anuran hosts and localities in Argentina. An Acad Bras Cienc. 2019;91. doi: 10.1590/0001-3765201920171028 31482992

[44] Grewal PS, Gaugler R, Selvan S. Host recognition by entomopathogenic nematodes: Behavioral response to contact with host feces. J Chem Ecol. 1993;19: 1219–1231. doi: 10.1007/BF00987382 24249139

[45] Spence KO, Lewis EE, Perry RN. Host-finding and invasion by entomopathogenic and plant-parasitic nematodes: Evaluating the ability of laboratory bioassays to predict field results. J Nematol. 2008;40: 93–98. 19259525 PMC2586539

[46] Lewis EE, Campbell J, Griffin C, Kaya H, Peters A. Behavioral ecology of entomopathogenic nematodes. Biological Control. 2006;38: 66–79. doi: 10.1016/j.biocontrol.2005.11.007

[47] Gang SS, Hallem EA. Mechanisms of host seeking by parasitic nematodes. Mol Biochem Parasitol. 2016;208: 23–32. doi: 10.1016/j.molbiopara.2016.05.007 27211240 PMC4993646

[48] Baiocchi T, Lee G, Choe DH, Dillman AR. Host seeking parasitic nematodes use specific odors to assess host resources. Sci Rep. 2017;7. doi: 10.1038/s41598-017-06620-2 28740104 PMC5524962

[49] Wells K, Clark NJ. Host Specificity in Variable Environments. Trends in Parasitology. Elsevier Ltd; 2019. pp. 452–465. doi: 10.1016/j.pt.2019.04.001 31047808

[50] Morand S, Legendre P, Lyell S, Jean G, Hugot P, Morand S, et al. Body size evolution of oxyurid (Nematoda) parasites: the role of hosts. Oecologia. 1996;107: 274–282. doi: 10.1007/BF00327912 28307314

[51] Poulin R, Morand S. Parasite body size and interspecific variation in levels of aggregation among nematodes. Journal of Parasitology. 2000;86: 642–647. doi: 10.1645/0022-3395(2000)086[0642:PBSAIV]2.0.CO;2 10864275

[52] Morand S, Sorci G. Determinants of Life-history Evolution in Nematodes. Parasitology Today. 1998;14: 193–196. doi: 10.1016/s0169-4758(98)01223-x 17040750

[53] Poulin R. The Evolution of Life History Strategies in Parasitic Animals. Adv Parasitol. 1996;37: 107–129. doi: 10.1016/s0065-308x(08)60220-1 8881599

[54] Victoria Herreras M, Montero F E., Marcogliese D J., Raga Antonio J, Balbuena J A. Phenotypic tradeoffs between egg number and egg size in three parasitic anisakid nematodes. Oikos. 2007;116: 1737–1747. doi: 10.1111/j.2007.0030-1299.16016.x

[55] Poulin R, Hamilton WJ. Egg size variation as a function of environmental variability in parasitic trematodes. Can J Zool. 2000;78: 564–569.

[56] Stearns SC. The Evolution of Life Histories. Oxford; 1992.

[57] Roff DA. Life History Evolution. Oxford University Press; 2002.

[58] Koprivnikar J, Randhawa HS. Benefits of fidelity: Does host specialization impact nematode parasite life history and fecundity? Parasitology. 2013;140: 587–597. doi: 10.1017/S0031182012002132 23343907

[59] Trejo-Meléndez VJ, Méndez-López TT, Contreras-Garduño J. The Coincidental Evolution of Virulence Partially Explains the Virulence in a Generalist Entomopathogenic. Acta Parasitol. 2023;68: 293–303. doi: 10.1007/s11686-023-00663-4 36806112 PMC10281897

[60] Hovestadt T, Thomas JA, Mitesser O, Schönrogge K. Multiple host use and the dynamics of host switching in host–parasite systems. Insect Conserv Divers. 2019;12: 511–522. doi: 10.1111/icad.12374

[61] Miranda VA, Navarro PD, Davidowitz G, Bronstein J, Stock SP. Effect of insect host age and diet on the fitness of the entomopathogenic nematode-bacteria mutualism. Symbiosis. 2013;61: 145–153. doi: 10.1007/s13199-013-0266-7

[62] Shapiro-Ilan D, Rojas MG, Morales-Ramos JA, Lewis EE, Tedders WL. Effects of host nutrition on virulence and fitness of entomopathogenic nematodes: Lipid-and protein-based supplements in Tenebrio molitor diets. J Nematol. 2008;40: 13–19. 19259513 PMC2586524

[63] Machovsky-Capuska GE, Raubenheimer D. The Nutritional Ecology of Marine Apex Predators. 2019. doi: 10.1146/annurev-marine-010318 31487471

[64] Meunier CL, Boersma M, Declerck SAJ, Laspoumaderes C. How sharp is the knife? Herbivore and carnivore sensitivity to resource stoichiometric quality. Oikos. 2023. doi: 10.1111/oik.09898

[65] Koppenhöfer AM, Fuzy EM. Attraction of four entomopathogenic nematodes to four white grub species. J Invertebr Pathol. 2008;99: 227–234. doi: 10.1016/j.jip.2008.05.003 18597774

[66] Allahverdipour HH, Karimi J. Nematodes Versus White Grubs: Long but Challenging Association. Ann Entomol Soc Am. 2021;114: 448–458. doi: 10.1093/aesa/saab016

[67] Katumanyane A, Slippers B, Wondafrash M, Malan AP, Hurley BP. Susceptibility of white grubs from forestry and sugarcane plantations in South Africa to entomopathogenic nematodes. BioControl. 2023;68: 155–167. doi: 10.1007/s10526-023-10185-7

[68] Jensen K, Mayntz D, Toft S, Clissold FJ, Hunt J, Raubenheimer D, et al. Optimal foraging for specific nutrients in predatory beetles. Proceedings of the Royal Society B: Biological Sciences. 2012;279: 2212–2218. doi: 10.1098/rspb.2011.2410 22237910 PMC3321704

[69] Haas W. Parasitic worms: strategies of host finding, recognition and invasion. Zoology. 2003;106: 349–364. doi: 10.1078/0944-2006-00125 16351919

[70] Chaisson KE, Hallem EA. Chemosensory behaviors of parasites. Trends in Parasitology. 2012. pp. 427–436. doi: 10.1016/j.pt.2012.07.004 22921895 PMC5663455

[71] Ramos-Rodríguez O, Campbell JF, Lewis EE, Shapiro-Ilan DI, Ramaswamy SB. Dynamics of carbon dioxide release from insects infected with entomopathogenic nematodes. J Invertebr Pathol. 2007;94: 64–69. doi: 10.1016/j.jip.2006.09.003 17054978

[72] Li XY, Cowles RS, Cowles EA, Gaugler R, Cox-Foster DL. Relationship between the successful infection by entomopathogenic nematodes and the host immune response. Int J Parasitol. 2007;37: 365–374. doi: 10.1016/j.ijpara.2006.08.009 17275827

[73] Guivier E, Lippens C, Faivre B, Sorci G. Plastic and micro-evolutionary responses of a nematode to the host immune environment. Exp Parasitol. 2017;181: 14–22. doi: 10.1016/j.exppara.2017.07.002 28733132

[74] Boff MIC, Wiegers GL, Smits PH. Influences of host size and host species on the infectivity and development of Heterorhabditis megidis (strain NLH-E87.3). BioControl. 2000.

[75] Wang Yi, Gaugler R, Cui Liwang. Variations in immune response of Popillia japonica and Acheta domesticus to Heterorhabditis bacteriophora and Steinernema species. J Nematol. 1994;26: 11–18. 19279863 PMC2619469

[76] Sorci G, Skarstein F, Morand S, Hugot JP. Correlated evolution between host immunity and parasite life histories in primates and oxyurid parasites. Proceedings of the Royal Society B: Biological Sciences. 2003;270: 2481–2484. doi: 10.1098/rspb.2003.2536 14667339 PMC1691528

[77] Babayan SA, Read AF, Lawrence RA, Bain O, Allen JE. Filarial parasites develop faster and reproduce earlier in response to host immune effectors that determine filarial life expectancy. PLoS Biol. 2010;8. doi: 10.1371/journal.pbio.1000525 20976099 PMC2957396

[78] Guivier E, Bellenger J, Sorci G, Faivre B. Helminth Interaction with the Host Immune System: Short-Term Benefits and Costs in Relation to the Infectious Environment. Am Nat. 2016;188: 253–263. doi: 10.1086/687149 27420789

[79] Huot L, George S, Girard PA, Severac D, Nègre N, Duvic B. Spodoptera frugiperda transcriptional response to infestation by Steinernema carpocapsae. Sci Rep. 2019;9. doi: 10.1038/s41598-019-49410-8 31501491 PMC6733877

[80] Stearns SC. Trade-Offs in Life-History Evolution. Funct Ecol. 1989;3: 259. doi: 10.2307/2389364

[81] Flatt T, Heyland A. Mechanisms of Life History Evolution. 2011.

[82] Tissenbaum HA. Using C. elegans for aging research. Invertebr Reprod Dev. 2015;59: 59–63. doi: 10.1080/07924259.2014.940470 26136622 PMC4464094

[83] Blaxter M, Koutsovoulos G. The evolution of parasitism in Nematoda. Parasitology. 2015;142: S26–S39. doi: 10.1017/S0031182014000791 24963797 PMC4413787

[84] Půža V, Mráček Z. Does scavenging extend the host range of entomopathogenic nematodes (Nematoda: Steinernematidae)? J Invertebr Pathol. 2010;104: 1–3. doi: 10.1016/j.jip.2010.01.002 20085768

[85] Sudhaus W. Preadaptive plateau in Rhabditida (Nematoda) allowed the repeated evolution of zooparasites, with an outlook on evolution of life cycles within Spiroascarida. Palaeodiversity (Stuttg). 2010;3: 117–130.

[86] Ramos-Rodríguez O., Campbell JF, Christen JM, Shapiro-Ilan DI, Lewis EE, Ramaswamy SB. Attraction behaviour of three entomopathogenic nematode species towards infected and uninfected hosts. Parasitology. 2007;134: 729–738. doi: 10.1017/S0031182006001880 17176490

[87] Blanco-Pérez R, Bueno-Pallero FÁ, Neto L, Campos-Herrera R. Reproductive efficiency of entomopathogenic nematodes as scavengers. Are they able to fight for insect’s cadavers? J Invertebr Pathol. 2017;148: 1–9. doi: 10.1016/j.jip.2017.05.003 28499929

[88] Canales-Lazcano J, Contreras-Garduño J, Cordero C. Strategic adjustment of copulatory plug size in a nematode. Curr Zool. 2019;65: 571–577. doi: 10.1093/cz/zoy067 31616488 PMC6784504

[89] Selvan S, Gaugler R, Lewis EE. Energy Biochemical Reserves of Nematodes. J Parasitol. 1993;79: 167–172.

[90] Selvan S, Gaugler R, Lewis EE. Biochemical Energy Reserves of Entomopathogenic Nematodes. Source: The Journal of Parasitology. 1993. Available: http://www.jstor.org URL:http://www.jstor.org/stable/3283503

[91] Patel MN, Wright DJ. Fatty Acid Composition of Neutral Lipid Energy Reserves in Infective Juveniles of Entomopathogenic Nematodes. Biochem Physiol. 1997. doi: 10.1016/s0305-0491(97)00057-6 9440227

[92] Patel MN, Wright DJ. Glycogen: its importance in the infectivity of aged juveniles of Steinernema carpocapsae. 2018. doi: 10.1017/S0031182097008780 9172428

[93] Wright DJ, Grewal PS, Stolinski M. Relative Importance of Neutral Lipids and Glycogen as Energy Stores in Dauer Larvae of Two Entomopathogenic Nematodes, Steinernema carpocapsae and Steinernema feltiae. Biochem Physiol. 1997. doi: 10.1016/s0305-0491(97)00165-x 9440220

[94] Moran MR. Changes in the Fat Content during Metamorphosis of the Mealworm, Tenebrio molitor Linnaeus. Journal of the New York Entomological Society. 1959;67: 213–216. Available: https://www.jstor.org/stable/25005700

[95] Rousell PG. Determination of glycogen content during the metamorphosis of the mealworm (Tenebrio molitor Linnaeus)*. New York Entomological Society. 1955;58: 107–110.

[96] Zuzarte-Luís V, Mota MM. Parasite Sensing of Host Nutrients and Environmental Cues. Cell Host Microbe. 2018;23: 749–758. doi: 10.1016/j.chom.2018.05.018 29902440

[97] Brooks DR, Agosta SJ. Children of time: The extended synthesis and major metaphors of evolution. Zoologia. 2012;29: 497–514.

[98] Viney M, Diaz A. Phenotypic plasticity in nematodes. Worm. 2012;1: 98–106. doi: 10.4161/worm.21086 24058831 PMC3670233

[99] Ruess L, Häggblom MM, García-Zapata EJ, Dighton J. Fatty acids of fungi and nematodes-possible biomarkers in the soil food chain? Soil Biol Biochem. 2002;34: 745–756. Available: www.elsevier.com/locate/soilbio

[100] Da Costa MS, Albuquerque L, Nobre MF, Wait R. The Identification of Fatty Acids in Bacteria. Methods in Microbiology. Elsevier Ltd; 2011. doi: 10.1016/B978-0-12-387730-7.00008-5

[101] De Carvalho CCCR, Caramujo MJ. Fatty acids as a tool to understand microbial diversity and their role in food webs of mediterranean temporary ponds. Molecules. 2014;19: 5570–5598. doi: 10.3390/molecules19055570 24786844 PMC6271346

[102] Ogier JC, Pagès S, Frayssinet M, Gaudriault S. Entomopathogenic nematode-associated microbiota: From monoxenic paradigm to pathobiome. Microbiome. 2020;8. doi: 10.1186/s40168-020-00800-5 32093774 PMC7041241

[103] Poinar GO, Hansen EL. Associations between nematodes and bacteria. Helminthology Abstracts. 1986;B 55: 61–79.

[104] McMullen JG, Peterson BF, Forst S, Blair HG, Stock SP. Fitness costs of symbiont switching using entomopathogenic nematodes as a model. BMC Evol Biol. 2017;17. doi: 10.1186/s12862-017-0939-6 28412935 PMC5392933

[105] Maher AMD, Asaiyah MAM, Brophy C, Griffin CT. An Entomopathogenic Nematode Extends Its Niche by Associating with Different Symbionts. Microb Ecol. 2017;73: 211–223. doi: 10.1007/s00248-016-0829-2 27543560

[106] Dahan D, Preston GM, Sealey J, King KC. Impacts of a novel defensive symbiosis on the nematode host microbiome. BMC Microbiol. 2020;20. doi: 10.1186/s12866-020-01845-0 32539750 PMC7296725

[107] Murfin KE, Dillman AR, Foster JM, Bulgheresi S, Slatko BE, Sternberg PW, et al. Nematode-Bacterium Symbioses-Cooperation and Conflict Revealed in the “Omics” Age. Biology Bulletin. 2012;223: 85–102. Available: http://www.biolbull.org/content/223/1/85.full. doi: 10.1086/BBLv223n1p85 22983035 PMC3508788

[108] Griffin CT. Perspectives on the behavior of entomopathogenic nematodes from dispersal to reproduction: Traits contributing to nematode fitness and biocontrol efficacy. J Nematol. 2012;44: 177–184. 23482343 PMC3578460

[109] Wolff GH, Riffell JA. Olfaction, experience and neural mechanisms underlying mosquito host preference. Journal of Experimental Biology. 2018;221. doi: 10.1242/jeb.157131 29487141 PMC7376831

[110] Shapiro-Ilan DI, Han R, Qiu X. Production of Entomopathogenic Nematodes. Mass Production of Beneficial Organisms: Invertebrates and Entomopathogens. Elsevier Inc.; 2013. pp. 321–355. doi: 10.1016/B978-0-12-391453-8.00010-8

[111] Labaude S, Griffin CT. Transmission success of entomopathogenic nematodes used in pest control. Insects. 2018;9. doi: 10.3390/insects9020072 29925806 PMC6023359

[112] Jaenike J. Suboptimal Virulence of an Insect-Parasitic Nematode. Evolution. 1996. Available: http://www.jstor.org URL:http://www.jstor.org/stable/2410694 Accessed:16-12-201515:58UTC doi: 10.1111/j.1558-5646.1996.tb03613.x 28565686

